# Alterations of the Innate Immune System in Susceptibility and Resilience After Social Defeat Stress

**DOI:** 10.3389/fnbeh.2018.00141

**Published:** 2018-07-13

**Authors:** Oliver Ambrée, Christina Ruland, Stefanie Scheu, Volker Arolt, Judith Alferink

**Affiliations:** ^1^Department of Psychiatry, University of Münster, Münster, Germany; ^2^Department of Behavioral Biology, University of Osnabrück, Osnabrück, Germany; ^3^Cluster of Excellence EXC 1003, Cells in Motion, University of Münster, Münster, Germany; ^4^Institute of Medical Microbiology and Hospital Hygiene, Heinrich Heine University of Düsseldorf, Düsseldorf, Germany

**Keywords:** chronic stress, social defeat, susceptibility, resilience, myeloid cells, major depressive disorder (MDD), monocytes, dendritic cells

## Abstract

Dysregulation of innate immune responses has frequently been reported in stress-associated psychiatric disorders such as major depression. In mice, enhanced circulating cytokine levels as well as altered innate immune cell numbers have been found after stress exposure. In addition, stress-induced recruitment of peripheral monocytes to the brain has been shown to promote anxiety-like behavior. However, it is yet unclear whether specific differences in the innate immune system are associated with stress susceptibility or resilience in mice. Utilizing chronic social defeat, a model of depression and stress vulnerability, we characterized peripheral and brain-invading myeloid cells in stress-susceptible and resilient animals. In all defeated animals, we found reduced percentages of CD11c^+^ dendritic cells (DCs) by flow cytometry in the spleen when compared to non-defeated controls. Exclusively in susceptible mice conventional DCs of the spleen showed up-regulated expression of MHC class II and co-stimulatory CD80 molecules pointing toward an enhanced maturation phenotype of these cells. Susceptible, but not resilient animals further exhibited an increase in inflammatory Ly6C^hi^ monocytes and higher numbers of spleen-derived CD11b^+^ cells that produced the proinflammatory cytokine tumor necrosis factor (TNF) upon lipopolysaccharide (LPS) stimulation. Increased percentages of peripheral CD45^hi^ CD11b^+^ cells immigrated into the brain of defeated mice, regardless of resilience or susceptibility. However, cellular infiltrates in the brain of susceptible mice contained higher percentages of CC chemokine receptor 2 (CCR2^+^) Ly6C^hi^ monocytes representing an inflammatory phenotype. Thus, we defined specific stress-related immune signatures involving conventional DCs and inflammatory Ly6C^hi^ monocytes in susceptible and resilient mice. Together, our findings suggest an impact of the innate immune system in vulnerability to stress-related disorders such as major depression.

## Introduction

Stressful experiences represent a major risk factor for mental illness such as major depressive disorder (MDD; Kessler et al., 2005; Krishnan and Nestler, 2008). However, physiological and psychological responses to stress vary and some individuals exhibit stress resilience (Krishnan and Nestler, 2008). Unraveling the underlying mechanisms of susceptibility and resilience is of major importance, due to the substantial burden that is associated with stress-associated MDD. In MDD patients, elevated levels of inflammatory markers including the proinflammatory cytokines tumor necrosis factor (TNF), interleukin (IL)-6, and IL-12 have been found in meta-analyses (Dowlati et al., 2010; Kohler et al., 2017). In addition to elevated cytokine levels, depressed patients show increased counts of neutrophils and monocytes that represent a prominent cellular source of these cytokines (Maes et al., 1992). However, it is an open question whether these immune changes are associated with stress vulnerability.

An established model to study stress susceptibility and resilience in mice is chronic social defeat. In this model, social avoidance behavior is induced in about one half of the animals while the others show social interaction comparable to controls (Krishnan et al., 2007). Susceptibility to social defeat stress has been shown to be mediated for instance by increased brain-derived neurotrophic factor (BDNF) signaling in mesolimbic dopamine (DA) pathways, enhanced firing rates of DA neurons, and activation of cortical projections from the prelimbic cortex to the nucleus accumbens (Berton et al., 2006; Krishnan et al., 2007; Cao et al., 2010; Vialou et al., 2014). Up to now, there is a lack of information on specific immune patterns especially involving myeloid cells associated with susceptibility and resilience in the chronic social defeat model. Only one study demonstrated that increased levels of IL-6 in mice predict and causally contribute to stress susceptibility (Hodes et al., 2014). However, innate immune changes specifically associated with stress-induced behaviors or resilience in this model are yet unresolved.

Other stress models in rodents revealed elevated levels of proinflammatory cytokines including TNF and IL-6, as well as the T helper cell differentiation cytokine IL-12 (Wohleb et al., 2011; Voorhees et al., 2013; Cheng et al., 2015). Acute and repeated stressors also increase neutrophil numbers (Engler et al., 2004a,b; Heidt et al., 2014; Lafuse et al., 2017). In addition, bone marrow-derived inflammatory CD11b^+^ Ly6C^hi^ monocytes increase in numbers after stress exposure (Wohleb et al., 2013; Heidt et al., 2014; Zheng et al., 2016; Lafuse et al., 2017). These cells have further been shown to immigrate into the brain of mice in response to repeated social disruption or foot shocks (Wohleb et al., 2011; Ataka et al., 2013). While these data point toward an important role of the innate immune response in stress and stress-associated behaviors, specific features of myeloid cells associated with stress vulnerability and resilience in the chronic social defeat model have not been studied.

The aim of this study was to identify specific innate immune cell profiles induced by chronic social defeat and to elucidate whether they are associated with stress vulnerability. We conducted flow cytometric analyses on *ex vivo* isolated spleen- and CNS-derived myeloid cells and intracellular cytokine staining after *in vitro* restimulation on peripheral myeloid cells of susceptible and resilient mice. Here we report specific innate immune signatures associated with stress vulnerability in these mice.

## Materials and Methods

### Mice and Housing Conditions

Male C57BL/6J mice were purchased at Charles River (Sulzfeld, Germany) at the age of 5 weeks and directly introduced into the experimental room. Animals were housed in groups of four in Makrolon type II-L cages (365 × 207 × 140 mm) for a period of 2 weeks before the experiments started. Male CD-1 mice of at least 3 months of age were used as resident animals for the social defeat paradigm and as partners in the social interaction test. Most of these mice had mating experience prior to the inclusion into this study. In addition, resident mice were checked for their aggressive behavior (latency to attack intruder should be less than 30 s). CD-1 males were housed singly in Makrolon type III cages (425 × 266 × 155 mm) until the experiment started. The experimental room was maintained at a temperature of 22 ± 2°C, humidity of 55 ± 10% and a 12 h:12 h light-dark cycle, with lights on at 6 am. Food and water were available *ad libitum*. This study was repeatedly performed in three independent cohorts with the addition of some additional parameters after the first cohort. Supplementary Table S1 gives an overview about sample sizes and analyzed parameters for each cohort. Within any cohort, control and social defeat mice were investigated and dissected at the same time. This study was performed in accordance with the regulations covering animal experimentation in Germany and the EU (European Communities Council Directive 2010/63/EU). The project was approved by the local authority (LANUV NRW) and the Animal Welfare Officer of the University of Münster. All efforts were made to minimize animal suffering and reduce the number of animals used.

### Social Defeat Paradigm

The social defeat paradigm applied in this study was based on the paradigm by Berton et al. (2006) with slight modifications in the setup of the social confrontation cage. All experimental mice were inserted into the cage of an aggressive, older and heavier CD-1 mouse for 10 min per day. After 10 min of direct physical contact, animals were separated by a perforated Plexiglas wall and kept on opposite sides of the same cage for 24 h. Thus, visual and olfactory contact between the animals was maintained, while physical contact and the danger of injuries were avoided. This procedure was repeated daily, every day with a novel, unfamiliar dominant CD-1 opponent. After the final confrontation, experimental mice where housed singly in Makrolon type II cages (267 × 207 × 140 mm). All experiments comprised 10 confrontations on subsequent days. The confrontations were performed in Makrolon type III cages (425 × 266 × 155 mm) which were inhabited by a CD-1 mouse for at least three days such that the cage could be considered its territory. The cages were modified in a way that a perforated Plexiglas wall could be introduced to separate the cage in two parts of equal size where on one side an additional water bottle and food pellets for the defeated mouse could be presented.

All defeat sessions have been observed by an experienced experimenter who carefully monitored the 10 min sessions and rated the degree of agonistic interactions on a scale between 0 and 3 based on the following definitions:

0. No agonistic encounters between the animals.
1. At least one agonistic encounter.
2. At least three bouts of the CD-1 mouse chasing the C57BL/6 intruder.
3. At least five bouts of chasing behavior or start of escalated fighting.

The experimenter terminated the sessions and separated the animals with the perforated Plexiglas divider as soon as escalated fighting occurred, even before 10 min passed. By this method, wounding of mice was successfully prevented to avoid potential influences of wounding on the innate immune response. However, few superficial scratches were equally found in susceptible and resilient animals. In a recent study, exclusively wounding, but not minor scratches, was associated with glucocorticoid resistance in splenocytes in a model of chronic social stress. Superficial scratches (considered non-wounded) did not induce further innate immune responses in addition to the stressor (Foertsch et al., 2017).

Control mice were housed in the same type of cage as experimental mice; however, two control mice were housed on the opposite sides of the perforated Plexiglas separation. To avoid effects induced by daily handling or cage change that was applied in experimental mice, control mice were daily handled and weighed. After weighing, control mice were put to the opposite compartments of the Plexiglas wall every day. After 10 days control mice were also housed singly.

### Social Interaction Test

One day after the last social defeat session, the social interaction test was conducted as described before (Ambrée et al. (2016), referred to as social exploration test). Briefly, it comprised two trials of 150 s each. During the first trial the enclosure in the social exploration box was empty; in the second trial (social exploration trial) an unfamiliar CD-1 mouse (male, between 3 months and 5 months of age) was present inside the exploration enclosure in the box. The time spent in the interaction zone, defined as the area surrounding the exploration enclosure 8 cm to each side, was recorded in both trials by ANY-maze tracking software (Stoelting, Dublin, Ireland). An interaction ratio was calculated as time spent in the interaction zone during the social exploration trial divided by the time spent in the zone during the first trial. Based on the interaction ratio, animals were defined as susceptible when the ratio was less than 0.5. In case the interaction ratio was greater than 0.5, animals were defined as resilient. The threshold of 0.5 was chosen to define resilient animals because the interaction ratio of control mice was in the same range with a mean around 1 and the smallest values starting around 0.5.

### Flow Cytometry of Splenocytes

After transcardial perfusion with ice-cold PBS, spleens were homogenized by digestion with collagenase type VIII and DNase I (Sigma-Aldrich, Munich, Germany) for 45 min at 37°C before preparation of a single-cell suspension. Cells were counted using a CASY cell counter (Roche, Mannheim, Germany). Following collection, cells were stored on ice for further use. Fluorescence staining was performed using the following antibodies purchased from Biolegend or BD Biosciences: PE-conjugated anti-mouse Ly6G (clone RB6-8C5), PerCP-Cy5.5-conjugated anti-mouse CD80 (clone 16-10A1), PE-Cy7-conjugated anti-mouse Ly6C (clone HK1.4), APC-conjugated anti-mouse MHC-II (clone M5/114.15.2), eFluor 450-conjugated anti-mouse CD11c (clone N418), BV510-conjugated anti-mouse CD11b (clone M1/70). Fc receptors were blocked with antibodies against mCD16/CD32 (Biolegend, San Diego, CA, USA). Samples were acquired on a FACSCanto II (BD Biosciences, East Rutherford, NJ, USA) flow cytometer and analyzed using FlowJo v10. Electronic gating included life gates to exclude debris and dead cells and additional gating strategies to discriminate doublets from single cells. The gating strategies to determine myeloid cell subsets are described in Figures 2A, 3A and Supplementary Figure S2.

**Figure 1:**
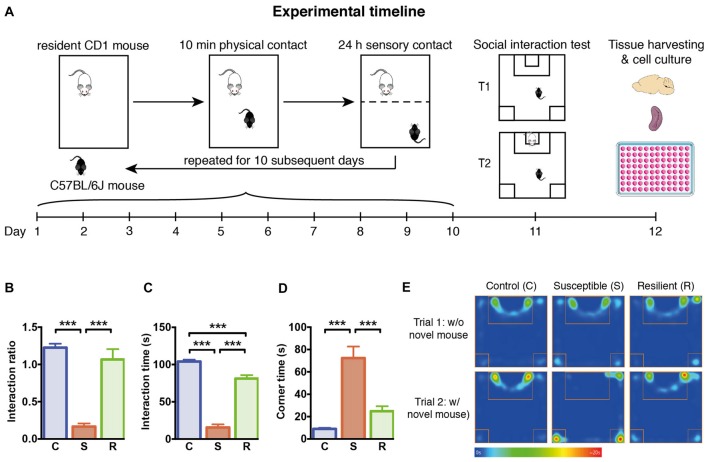
Experimental timeline and social behavior. **(A)** Graphical representation of the experimental timeline. C57BL/6J mice were subjected to 10 min daily confrontation with CD-1 mice for 10 subsequent days. In between, mice were housed in the same cages separated by a perforated Plexiglas wall from the dominant and aggressive conspecific. At day 11, mice were tested in the social interaction test, before they were sacrificed 1 day later for tissue harvesting and cellular analysis. **(B)** Significantly reduced interaction ratio in susceptible mice calculated as time spent in the interaction zone in trial 2 in relation to the time in trial 1. **(C)** Significantly decreased time spent in the interaction zone during the 2nd trial of the social interaction test in susceptible mice. **(D)** Significantly increased time spent in the corners opposite to the interaction zone during the 2nd trial of the social interaction test in susceptible mice. **(E)** Mean heat map of the groups’ center points for control, susceptible and resilient mice in the 1st and 2nd trial of the social interaction test. Bar graphs represent mean + SEM. T1, trial 1; T2, trial 2; C, control; S, susceptible; R, resilient. n_C_ = 16, n_S_ = 15, n_R_ = 15. ***p < 0.001.

**Figure 2:**
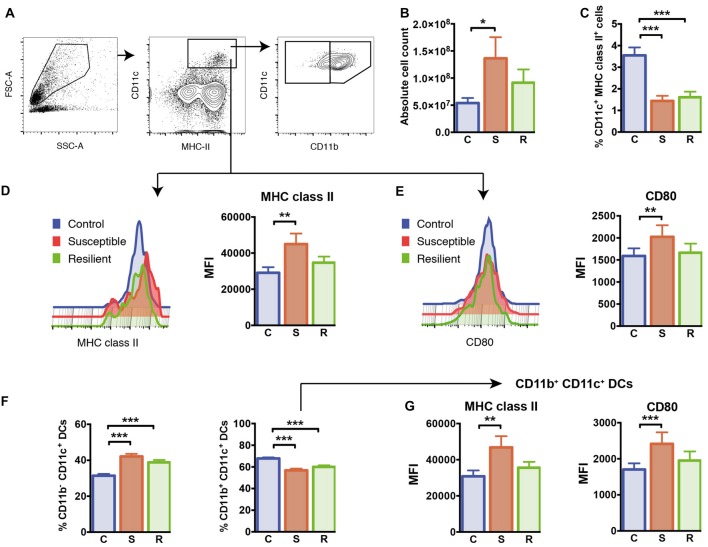
Cellularity and dendritic cells (DCs) in the spleen. **(A)** Gating of CD11c^+^ MHC class II^+^ DCs and CD11b^+^/CD11b^−^ DC subsets. **(B)** The absolute cell numbers isolated from the spleens of experimental mice. **(C)** Reduced percentages of CD11c^+^ MHC class II^+^ DCs in mice subjected to social defeat. **(D)** Elevated expression of MHC class II molecules in susceptible mice pregated for CD11c^+^ MHC class II^+^ DCs. **(E)** Elevated expression of CD80 molecules in susceptible mice pregated for CD11c^+^ MHC class II^+^ DCs. **(F)** Increased percentages of the CD11b^−^ CD11c^+^ DC subset and decreased percentages of the CD11b^+^ CD11c^+^ DC subset in socially defeated mice. **(G)** Elevated expression of MHC class II molecules and CD80 molecules in CD11b^ +^ CD11c^+^ DCs of susceptible mice. Bar graphs represent mean + SEM. C, control; S, susceptible; R, resilient; MFI, mean fluorescence intensity. Absolute cell count: n_C_ = 16, n_S_ = 15, n_R_ = 15, all other graphs in this Figure: n_C_ = 10, n_S_ = 11, n_R_ = 9. *p < 0.05, **p < 0.01, ***p < 0.001.

**Figure 3:**
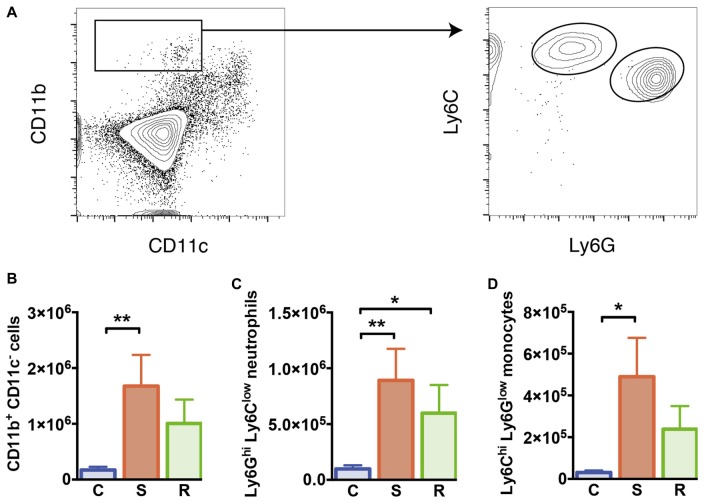
Monocytes and neutrophils in the spleen. **(A)** Gating of CD11b^+^ CD11c^−^ myeloid cells, Ly6C^hi^ Ly6G^low^ inflammatory monocytes, and Ly6G^hi^ Ly6C^low^ neutrophils. **(B)** Increased numbers of CD11b^+^ CD11c^−^ myeloid cells in susceptible mice. **(C)** Increased numbers of Ly6G^hi^ Ly6C^low^ neutrophils in socially defeated mice. **(D)** Increased numbers of Ly6C^hi^ Ly6G^low^ inflammatory monocytes in susceptible mice. Bar graphs represent mean + SEM. C, control; S, susceptible; R, resilient. n_C_ = 10, n_S_ = 11, n_R_ = 9. *p < 0.05, **p < 0.01.

### *In Vitro* Stimulation of Splenocytes With LPS

**Figure 4:**
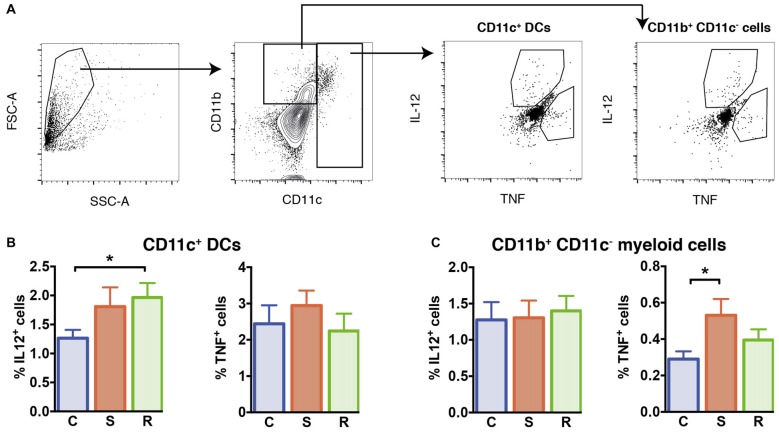
Cytokine production in LPS stimulated isolated splenocytes. **(A)** Gating of interleukin (IL)-12^+^ and tumor necrosis factor (TNF)^+^ CD11c^+^ DCs and CD11b^+^ CD11c^−^ myeloid cells. **(B)** Significantly increased percentages of IL-12^+^ cells in resilient mice among the CD11c^+^ DCs and similar percentages of TNF^+^ DCs. **(C)** Similar percentages of IL-12^+^ cells and increased percentages of TNF^+^ cells among the CD11b^+^ CD11c^−^ myeloid cells in susceptible mice. Bar graphs represent mean + SEM. C, control; S, susceptible; R, resilient. n_C_ = 15, n_S_ = 14, n_R_ = 14. *p < 0.05.

Splenocytes (six wells/animal containing 5 × 10^5^ cells/100 μl) were cultured in DMEM supplemented with 10% (v/v) heat-inactivated FCS, L-glutamine, penicillin/streptomycin, 2-mercaptoethanol (all from Gibco) and stimulated for 10 h with 100 ng/ml *Escherichia coli* Lipopolysaccharide (LPS) Serotype O127:B8 (Sigma Aldrich, St. Louis, MO, USA) plus Monensin and Brefeldin A (Biolegend, San Diego, CA, USA). Fluorescence staining was performed using the following antibodies purchased from Biolegend or BD Biosciences: PE-conjugated anti-mouse IL-12 p40/p70 (clone C15.6), biotinylated anti-mouse CD11b (clone M1/70) and Streptavidin conjugated to PerCP/Cy5.5, APC-conjugated anti-mouse TNF α (clone MP6-XT22), eFluor 450-conjugated anti-mouse CD11c (clone N418). Fc receptors were blocked with antibodies against mCD16/CD32 (Biolegend, San Diego, CA, USA). Intracellular staining was performed according to the manufacturer’s instruction using the Fixation/Permeabilization kit (BD Cytofix/Cytoperm). Samples were acquired on a FACSCanto II (BD Biosciences, East Rutherford, NJ, USA) flow cytometer and analyzed using FlowJo v10 (TreeStar™). Electronic gating included life gates to exclude debris and dead cells and additional gating strategies to discriminate doublets from single cells. The gating strategies to determine cytokine producing myeloid cell subsets are described in Figure 4A and Supplementary Figure S2.

### Flow Cytometry of CNS Mononuclear Cells

**Figure 5:**
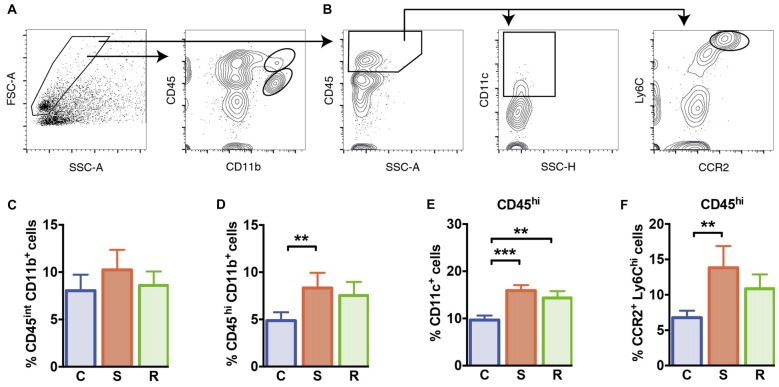
Microglia and peripheral myeloid cells in the brain. **(A)** Gating of CD45^int^ CD11b^+^ microglia and CD45^hi^ CD11b^+^ brain invading myeloid cells. **(B)** Gating of CD11c^+^ DCs and CCR2^+^ Ly6C^hi^ inflammatory monocytes of CD45hi brain invading peripheral cells. **(C)** Similar percentages of CD45^int^ CD11b^+^ microglia. **(D)** Significantly increased percentages of CD45^hi^ CD11b^+^ brain invading myeloid cells in susceptible mice. **(E)** Increased percentages of CD11c^+^ DCs among CD45^hi^ brain invading immune cells in socially defeated mice. **(F)** Significantly increased percentages of CCR2^+^ Ly6C^hi^ inflammatory monocytes among CD45^hi^ brain invading immune cells in susceptible animals. Bar graphs represent mean + SEM. C, control; S, susceptible; R, resilient. CD45^int/hi^ CD11b^+^: n_C_ = 16, n_S_ = 13, n_R_ = 15. CD45^hi^ CD11c^+^, CD45^hi^ CCR2^+^ Ly6C^hi^: n_C_ = 10, n_S_ = 9, n_R_ = 9. **p < 0.01, ***p < 0.001.

Mice were perfused with ice-cold PBS to remove leucocytes from intracerebral blood vessels. Brains were homogenized by manual disruption and incubation in Collagenase/Dispase, and DNase I at 37°C for 45 min each (Roche Applied Science, Mannheim, Germany). Mononuclear CNS cells were enriched by gradient centrifugation of 30% and 70% Percoll (GE Healthcare, Ltd., Buckinghamshire, UK). Enriched cells were collected from the 30%/70% interface of the Percoll gradient after centrifugation at 921× *g* for 25 min at room temperature. Cells were counted using a CASY cell counter (Roche, Mannheim, Germany). Following collection, cells were stored on ice for further use. Fluorescence staining was performed using the following antibodies purchased from Biolegend or BD Biosciences: PE-conjugated anti-mouse CCR-2 (clone 475301), PE-Cy7-conjugated anti-mouse Ly6C (clone HK1.4), APC-Cy7-conjugated anti-mouse CD45 (clone 30-F11), eFluor 450-conjugated anti-mouse CD11c (clone N418), BV510-conjugated anti-mouse CD11b (clone M1/70). Fc receptors were blocked with antibodies against mCD16/CD32 (Biolegend, San Diego, CA, USA). Samples were acquired on a FACSCanto II (BD Biosciences, East Rutherford, NJ, USA) flow cytometer and analyzed using FlowJo v10 (TreeStar™). Electronic gating included life gates to exclude debris and dead cells and additional gating strategies to discriminate doublets from single cells. The gating strategies to determine intracerebral leucocyte subsets are described in Figures 5A,B and Supplementary Figure S2.

### Statistics

For the statistical analysis, data obtained in three independent cohorts were combined and analyzed by analysis of covariance (ANCOVA) with stress phenotype as fixed factor and cohort as covariate. In case of significant effects of the stress phenotype, Bonferroni posthoc tests were calculated. To support findings from the ANCOVA analysis, Pearson correlations were calculated between immune parameters that were differentially affected in susceptible and resilient mice and measures of social avoidance. Immune parameters were transformed to percent of the control mean within each cohort to correct for inter cohort variability. The null-hypothesis was rejected for *p* < 0.05. All analyzes were calculated with SPSS 24 (IBM).

## Results

### Social Defeat Stress Is Associated With Social Avoidance in Susceptible Mice

To investigate the effects of stress on the innate immune response, we subjected 7 week old C57BL/6 mice to 10 days of social defeat stress (Figure 1A). One day later, the exploration of an unfamiliar social partner was assessed in the social interaction test. Based on the definition of susceptible and resilient mice, social defeat stress resulted in a markedly reduced interaction ratio in susceptible mice compared to controls (*p* < 0.001) and resilient mice (*p* < 0.001, Figure 1B). The reduced exploration of the conspecific was associated with increased time spent in the opposite corners of the apparatus (Figures 1C–E), with susceptible mice spending significantly less time in the interaction zone and more time in the corners as controls (*p* < 0.001) and resilient animals (*p* < 0.001).

With regard to physiological stress responses, body weight was similar between the groups at the beginning and the end of the experiment. At the time of tissue harvesting, basal corticosterone levels were increased exclusively in susceptible mice compared to resilient or control animals (C vs. S: *p* < 0.01; S vs. R: *p* < 0.01, Supplementary Figure S1). In contrast, the total number of thymocytes was significantly reduced in both defeated groups when compared to controls (C vs. S: *p* < 0.05; C vs. R: *p* < 0.01). These data indicate that a chronic stress response occurred in all defeated mice while corticosterone levels were only affected in susceptible mice.

To exclude whether the susceptible or resilient phenotype could be influenced by heterogeneity in fighting behavior during sessions, intensity of agonistic interactions was assessed by daily scoring. Susceptible and resilient mice showed equivalent numbers of agonistic encounters during defeat sessions indicating that behavioral outcome does not result from variations in aggressive behavior (Supplementary Figure S1E). These findings support the notion that the resilient phenotype does not result from fewer aversive experiences during the 10-day stress phase.

### Altered Phenotype of Conventional Dendritic Cells in Stress Susceptible Mice

To analyze the effects of social defeat stress on the splenic immune cell composition and its potential association with stress susceptibility or resilience, we first quantified total numbers of mononuclear cells in the spleen. There was a significant difference in the absolute cell number per spleen between the groups (Figure 2B, *F*_(2,41)_ = 4.47, *p* = 0.018). In contrast to resilient mice, susceptible animals showed a significant increase in cellularity with higher total cell numbers in their spleens when compared to control mice (*p* = 0.015).

To study whether cells of the innate immune system are specifically affected by susceptibility or resilience to stress, we first focused on CD11c^+^ dendritic cells (DCs). Both stress groups had markedly reduced percentages of CD11c^+^ DCs in the spleen compared to controls (Figure 2C, C vs. S: *p* < 0.001; C vs. R: *p* < 0.001). The expression levels of cell surface molecules associated with antigen presentation and co-stimulation, MHC class II and CD80, were increased on DCs of susceptible mice compared to controls (Figures 2D,E, MHC class II: *p* = 0.003; CD80: *p* = 0.017).

We further quantified proportions of conventional CD11b^+^ CD11c^+^ DCs as well as CD11b^−^ CD11c^+^ DCs in this model. While percentages of CD11b^−^ CD11c^+^ DCs were equivalently increased in both stress groups (C vs. S: *p* < 0.001; C vs. R: *p* < 0.001), the subset of conventional CD11b^+^ CD11c^+^ DCs was reduced in these animals (Figure 2F, C vs. S: *p* < 0.001; C vs. R: *p* < 0.001). As shown for the total population of CD11c^+^ DCs, surface expression levels of MHC class II and CD80 molecules were increased in conventional CD11b^+^ CD11c^+^ DCs of susceptible mice, but not in resilient and control animals (Figure 2G, MHC class II: *p* = 0.008; CD80: *p* = 0.001). With regard to the CD11b^−^ CD11c^+^ DC subset, these cells showed higher surface expression of MHC class II in the spleen of susceptible mice compared to control mice (*p* = 0.001). These data indicate that equally reduced proportions of conventional DCs occur in stress resilient as well as in susceptible mice. However, levels of MHC class II and co-stimulatory CD80 molecules are upregulated on conventional DCs exclusively in the spleen of susceptible mice.

### Inflammatory Ly6C^hi^ Monocytes Prominently Increase in Stress Susceptible Mice

As mentioned before, spleen cellularity increased in susceptible mice compared to control animals. As a result absolute numbers of DCs were not altered (*F*_(2, 26)_ = 0.09, *p* = 0.913, Supplementary Table S2). These data indicate that other non-DC cell populations expand in stress susceptibility. Accordingly, susceptible animals showed significantly elevated numbers of CD11b^+^ myeloid cells that were negative for CD11c in the spleen compared to controls (Figure 3B, *p* = 0.006). A mild but non-significant increase in these cells was also detectable in resilient mice. About half of these cells could be identified as Ly6G^hi^ Ly6C^low^ neutrophils, which showed significantly higher numbers in susceptible and resilient mice (Figure 3C, C vs. S: *p* = 0.004; C vs. R: *p* = 0.048). The Ly6C^hi^ Ly6G^low^ subset of CD11b^+^ CD11c^−^ myeloid cells has been classified before as inflammatory monocytes (Gordon and Taylor, 2005). Susceptible mice presented elevated numbers of this cell type compared to controls (Figure 3D, *p* = 0.019). Together, these data indicate stress-induced alterations of the innate immune compartment in all defeated animals. However, a substantial increase in inflammatory Ly6C^hi^ monocytes specifically occurred in susceptible mice.

### Enhanced Percentages of TNF-Producing CD11b^+^ Cells in Stress Susceptible Mice

We explored the functional capacity of myeloid cells in stress susceptible and resilient mice and determined the capacity of these cells to produce IL-12 and TNF in response to Toll-like receptor ligand LPS. For this, we performed intracellular cytokine staining of splenic myeloid cells upon *in vitro* LPS stimulation and assessed the percentage of CD11c^+^ DCs and CD11b^+^ cells negative for CD11c that produced IL-12 and TNF. Resilient mice exhibited a higher percentage of DCs that produced IL-12 when compared to controls (Figure 4B, *p* = 0.039). In susceptible mice a mild but not significant increase was observed. The percentages of TNF-producing DCs were similar between all groups (*F*_(2,38)_ = 0.21, *p* = 0.811).

With regard to CD11b^+^ myeloid cells, equivalent percentages of IL-12 producing cells were found in stressed animals and controls (*F*_(2,38)_ = 0.40, *p* = 0.675). However, in the spleen of susceptible mice higher percentages of TNF-producing CD11b^+^ cells were found (Figure 4C, *p* = 0.033 vs. C). These data indicate that stress susceptibility is associated with increased percentages of CD11b^+^ cells producing the inflammatory cytokine TNF, while resilience is associated with higher percentages of DCs producing the T cell differentiation cytokine IL-12.

### Increased Brain Recruitment of Inflammatory CCR2^+^ Ly6C^hi^ Monocytes in Susceptibility

Finally, we quantified microglia and brain infiltrating peripheral myeloid cells in the brain of these animals. Social defeat stress did not affect the percentage of CD45^int^ CD11b^+^ microglia compared to controls (Figure 5C, *F*_(2,39)_ = 1.17, *p* = 0.322). However, percentages of CNS invading CD45^hi^ CD11b^+^ myeloid cells that comprise peripheral monocytes/macrophages were significantly increased in susceptible mice (Figure 5D, *p* = 0.001). Resilient mice showed a mild increase in CD45^hi^ CD11b^+^ cell percentages, that did not reach statistical significance (*p* = 0.053 vs. C). Among CD45^hi^ CNS infiltrates, DC percentages were significantly higher in defeated mice (Figure 5E, C vs. S: *p* < 0.001; C vs. R: *p* = 0.004). Percentages of infiltrating CC chemokine receptor 2 (CCR2^+^) Ly6C^hi^ inflammatory monocytes were elevated in susceptible mice (Figure 5F, *p* = 0.007 vs. C) again hinting toward an activated immune status in susceptible defeated mice.

### Innate Immune Parameters Correlate With Measures of Social Avoidance

To support the findings that specific parameters of the innate immune system are associated with stress susceptibility or resilience, we calculated correlations between the respective immune parameters and measurements of social avoidance. With the exception of percentages of splenic Ly6G^hi^ neutrophils and stimulated IL-12-producing DCs, all other immune parameters differentially affected in susceptible or resilient mice showed significant correlations with the interaction ratio and the interaction time (Supplementary Table S3). Significant correlations include expression levels of MHC class II and CD80 on DCs, percentages and numbers of CD11b^+^ monocytes as well as numbers of Ly6C^hi^ inflammatory monocytes. Furthermore, percentages and numbers of stimulated TNF producing CD11b^+^ monocytes correlated significantly with social avoidance. This also holds true for the percentages of CD45^hi^ CD11b^+^ brain invading peripheral myeloid cells and percentages of CCR2^+^ Ly6C^hi^ CD45^hi^ brain invading inflammatory monocytes.

## Discussion

In this study, we characterized alterations in the innate immune system after chronic social defeat stress. Specifically, we determined the phenotype and cytokine producing capacity of various subsets of innate immune cells in stress susceptible and resilient mice. Our data indicate that changes in the myeloid cell compartment involving DCs as well as peripheral and brain invading inflammatory Ly6C^hi^ monocytes are specifically associated with stress susceptibility and resilience based on stress-induced social avoidance. Quantification of DCs in the spleen revealed reduced percentages of these cells in defeated mice, regardless of susceptibility or resilience. Susceptible mice exclusively showed an enhanced maturation phenotype of DCs with elevated expression of MHC class II and co-stimulatory CD80 molecules. In addition, they exhibited higher percentages of TNF-producing CD11b^+^ cells in the spleen. Susceptibility to stress, but not resilience, was further associated with an increase in inflammatory Ly6C^hi^ monocytes in the spleen and enhanced recruitment of these cells to the brain.

DCs process and present antigens to T cells and therefore represent the main interface between the innate and the adaptive immune response (Takagi et al., 2011; Fukaya et al., 2012; Steinman, 2012). Alterations in the maturation phenotype of splenic DCs characterized by increased levels of MHC class I, CD80 and CD44 molecules have previously been reported in mice after 6 days of repeated social disruption stress (Powell et al., 2009). This altered DC phenotype may be caused by recognition of damage-associated molecular pattern molecules (DAMPs). DAMPs act as endogenous danger signals and alert the innate immune system in response to stress (Fleshner et al., 2017; Franklin et al., 2017). Our data now demonstrate that only susceptibility but not resilience to social stress impacts DC maturation, at least at the level of surface marker expression. These phenotypic alterations specifically occurred in the subset of conventional CD11b^+^ CD11c^+^ DCs. In susceptible mice, however, phenotypic changes did not translate to an augmented IL-12 production by these cells. Instead, resilient, but not susceptible animals, displayed an increased proportion of IL-12-producing cells in spleen-derived DCs upon LPS stimulation. IL-12 is known to control the differentiation of naïve CD4^+^ T cells into T helper 1 cells (Trinchieri, 1995; Watford et al., 2003), which play an important role in adaptive immune responses and have also been implicated in stress responses (Dong and Flavell, 2001; Watford et al., 2003). This “split phenotype” of DCs in resilient and susceptible animals may reflect yet unknown distinct functional properties of these cells associated with differences in behavioral responses to social stress.

In our study, absolute numbers of DCs were not affected by social defeat. However, since numbers of other non-DC cell populations such as neutrophils and other CD11b^+^ cells increased in the spleen, DC proportions were reduced in both, susceptible and resilient mice. This relative reduction in the DC compartment might compromise their antigen presentation or immunoregulatory properties (Abe et al., 2003; Strother et al., 2016). On the other side, it is possible that enhanced DC maturation augments T cell responses in susceptible mice (Takagi et al., 2011; Fukaya et al., 2012). This has been shown in a model of repeated social disruption. Herein, adoptive transfer of DCs from stressed mice that displayed an enhanced maturation phenotype conferred enhanced adaptive immunity against influenza A virus to recipient animals (Powell et al., 2011). In addition, a plethora of studies supports a role for various CD4^+^ T cell subsets, particularly pathogenic Th17 cells and T regulatory cells, in stress-induced behaviors in mice (Kim et al., 2012; Beurel et al., 2013; Hong et al., 2013). Furthermore, altered numbers or ratios of these T cell subsets are associated with stress-relevant disorders such as MDD (Chen et al., 2011; Grosse et al., 2016). In this context, we further observed a larger proportion of DCs among peripheral myeloid cells that invaded the brain in both, susceptible and resilient mice. In models of neuroinflammation and CNS autoimmunity, we and others have previously demonstrated a prominent role of DCs in the re-activation of primed T cells upon CNS entry and in local maintenance of Th17 cells (Goverman, 2009; Ransohoff and Cardona, 2010; Poppensieker et al., 2012; Ruland et al., 2017; Scheu et al., 2017). These findings underscore the need to characterize DC-mediated T cell responses in stress susceptibility and resilience after social defeat in future studies. In addition, the effect of CNS invading DCs on CNS resident microglia, astrocytes, and neurons in this model is yet unknown.

In contrast to DCs, neutrophil numbers were equally increased in susceptible and resilient animals. Our findings are in accordance with earlier studies in humans and rodents demonstrating stress-induced enhancement of neutrophil counts (Engler et al., 2004a,b; Heidt et al., 2014; Lafuse et al., 2017). Neutrophils orchestrate the early immune response and react immediately to a pathogenic assault via utilizing various cytotoxic mechanisms (Kobayashi and DeLeo, 2009). Functionally, the increase in neutrophil numbers could serve as adaptive response to stress. This neutrophil-mediated response may prepare the organism for dangerous situations which involve an increased risk for injuries or infections (Dhabhar, 2013). Until now, the function of neutrophils and their potential involvement in stress-induced behavioral changes are not yet fully understood.

In our study, specifically susceptible mice exhibited an increased proportion of CD11b^+^ monocytes that produced proinflammatory TNF after *in vitro* stimulation with LPS. It has frequently been shown that stress exposure results in activation of an inflammatory immune response in mice. For instance, increased blood levels of pro-inflammatory cytokines such as TNF, IL-1β, or IL-6 have been reported after chronic mild stress, social defeat or learned helplessness (Grippo et al., 2005; Hodes et al., 2014; Yang et al., 2015). Our data are further in accordance with earlier studies demonstrating that social defeat stress is associated with higher TNF production by LPS stimulated cultured splenocytes (Kinsey et al., 2008). Various studies have further reported enhanced levels of TNF and other proinflammatory cytokines in the blood and/or cerebrospinal fluid of patients suffering from MDD (Dantzer et al., 2008). Mechanistically, it is thought that peripheral proinflammatory cytokines reach the brain via distinct humoral, neural, and cellular pathways and locally induce detrimental microglia responses (Reyes et al., 1999; Banks, 2005; Capuron and Miller, 2011). Consecutively, activated microglia release proinflammatory cytokines and neurotoxic factors that negatively affect CNS cell functions and thus trigger anxiety- and depression-like behaviors in mice (Reader et al., 2015; Lehmann et al., 2016; Ramirez et al., 2017; Stein et al., 2017). In accordance, earlier studies demonstrated that treatment with TNF induced a depressive-like state in mice (Kaster et al., 2012). TNF application further mediates activation of microglia (Qin et al., 2007), which may result in a self-perpetuating loop of neuroinflammatory disturbance resulting in depression-like behavior. In murine stress models, TNF has also been reported to upregulate indoleamine 2,3-dioxygenase (IDO) implicated as biological mediator of inflammation-related mood disorders (Liu et al., 2015). TNF release has finally been functionally involved in the trafficking of immune cells into the brain during social defeat stress that may locally contribute to production of TNF and other proinflammatory cytokines (Wohleb and Delpech, 2017). In accordance, a recent study determined that upregulation of TNF was associated with higher numbers of Iba-1^+^ microglia in the prefrontal cortex of stress susceptible animals (Couch et al., 2013). Interestingly, we also observed enhanced migration of a subset of CD11b^+^ Ly6C^hi^ monocytes into the brain of susceptible animals, albeit the role of TNF in this process is still unclear.

In addition, CD11b^+^ Ly6C^hi^ monocytes have been shown before to express an inflammatory functional phenotype (Gordon and Taylor, 2005). We demonstrated that numbers of this monocyte subset increased in the spleen and brain of susceptible mice. This is in line with previous studies showing enhanced numbers of CD11b^+^ Ly6C^hi^ inflammatory monocytes after stress exposure (Wohleb et al., 2013; Heidt et al., 2014; Zheng et al., 2016; Lafuse et al., 2017). Bone marrow-derived myeloid cells have also been shown before to enter the brain of mice that have been stressed by repeated social disruption or foot shocks (Wohleb et al., 2011; Ataka et al., 2013). The recruitment of circulating CD11b^+^ Ly6C^hi^ monocytes into the CNS requires signaling via fractalkine receptor CX_3_CR1 and CC chemokine receptor 2 (CCR2), the cognate receptor for CC chemokine ligand 2 (CCL2; Wohleb et al., 2013). Deficiency in CX_3_CR1 or CCR2, did not affect the increase of Ly6C^hi^ monocytes in the blood after social disruption stress, but prevented brain infiltration of CD45^hi^ CD11b^+^ macrophages and the development of stress-induced anxiety (Wohleb et al., 2013). It was further shown, that treatment of mice with the natural ginsenoside Rg1 reduced the proinflammatory potential of Ly6C^hi^ monocytes and suppressed their recruitment to the brain via inhibition of CCL2-induced signaling pathways after LPS challenge (Zheng et al., 2016). In consequence, Rg1 treatment resulted in antidepressant effects and reduced social avoidance and anxiety-like behavior after social defeat stress (Zheng et al., 2016). These and other findings in mice showing brain infiltration of Ly6C^hi^ or CD11b^+^ monocytes after 10 days of social defeat stress suggest that brain invasion of these cells is necessary to induce stress-induced behavioral effects (Zheng et al., 2016; Menard et al., 2017). However, these studies did not distinguish between susceptible and resilient mice regarding peripheral myeloid cells in the brain. In our experiment resilient and susceptible animals showed a similar proportion of CD45^hi^ CD11b^+^ peripheral myeloid cells that immigrated into the brain. In contrast, only susceptible mice exhibited enhanced recruitment of Ly6C^hi^ inflammatory monocytes to the brain. These data suggest that brain invading Ly6C^hi^ monocytes may differentially affect behavior in response to stress. As suggested before, these cells may represent an effector cell type involved in promoting behavioral effects in susceptible animals (Wohleb et al., 2013; Zheng et al., 2016).

It is well established, that manipulation of the immune response before induction of a stress response, e.g., by depletion or adoptive transfer of Th17 cells (Beurel et al., 2013), adoptive T cell transfers from stress-exposed donor mice into non-stressed recipients (Brachman et al., 2015), stressor-combined LPS application (Couch et al., 2016), or immunization with heat-killed preparations of microorganisms (Reber et al., 2016) may alter stress vulnerability. Also exposure of mice to distinct gut pathobionts may affect individual variability in susceptibility to stress-associated pathologies (Langgartner et al., 2017). In sum these findings underscore the essential role of altered immune responses for the behavioral reaction to stress. However, it is an open question, whether immune signatures observed in our study harbor predictive potential for stress susceptibility or resilience. We performed endpoint analyses associated with the social avoidance phenotype after stress exposure. Future studies will determine whether a differential immune reactivity before or immediately after onset of social defeat can predict stress vulnerability.

In summary, our findings demonstrate that innate immune alterations occur in response to stress and that stress susceptibility in this model is specifically associated with distinct immune patterns. Susceptible, but not resilient mice exhibit an enhanced DC maturation phenotype, activation of peripheral monocytes, and increased brain recruitment of inflammatory monocytes. Further studies will unravel the influence of the observed DC signature on T cell responses, and the impact of an altered innate immune response on neuronal functions. Together, our findings contribute to a better understanding of immune alterations in stress susceptibility and resilience and their relation to stress-induced behavioral changes.

## Author Contributions

OA, SS, VA and JA designed the study and the experiments. OA and CR performed the experiments. OA and JA analyzed the data and wrote the first draft of the manuscript. All authors contributed to the revision of the manuscript, read and approved the submitted version.

## Conflict of Interest Statement

VA is member of advisory boards and/or gave presentations for the following companies Astra-Zeneca, Eli Lilly, Janssen-Organon, Lundbeck, Otsuka, Servier and Trommsdorff. The remaining authors declare that the research was conducted in the absence of any commercial or financial relationships that could be construed as a potential conflict of interest.
