# Supplementary material for: Alterations of the Innate Immune System in Susceptibility and Resilience After Social Defeat Stress
Source: Front Behav Neurosci. 2018 Jul 13;12:141. doi: 10.3389/fnbeh.2018.00141 (PMC6053497; doi:10.3389/fnbeh.2018.00141)
Supplement: Supplementary file 1 [file Table_1.PDF]

**Supplementary Table 1.** Overview of cohorts and sample sizes. C: control, S: susceptible, R: resilient.

| Spleen cells unstimulated* | Sample size behavior | | | Parameters flow cytometry | Sample size flow cytometry | | |
|---|---|---|---|---|---|---|---|
| | C | S | R | | C | S | R |
| Cohort 1 | 6 | 4 | 6 | | -[a] | -[a] | -[a] |
| Cohort 2 | 5 | 6 | 4 | CD11b, CD11c, CD80, Ly6C, Ly6G, MHC-II | 5 | 6 | 4 |
| Cohort 3 | 5 | 5 | 5 | CD11b, CD11c, CD80, Ly6C, Ly6G, MHC-II | 5 | 5 | 5 |
| Total | 16 | 15 | 15 | | 10 | 11 | 9 |

| LPS stimulated spleen cells* | Sample size behavior | | | Parameters flow cytometry | Sample size flow cytometry | | |
|---|---|---|---|---|---|---|---|
| | C | S | R | | C | S | R |
| Cohort 1 | 6 | 4 | 6 | CD11b, CD11c, IL-12, TNF | 5[b] | 3[b] | 6 |
| Cohort 2 | 5 | 6 | 4 | CD11b, CD11c, IL-12, TNF | 5 | 6 | 4 |
| Cohort 3 | 5 | 5 | 5 | CD11b, CD11c, IL-12, TNF | 5 | 5 | 4[c] |
| Total | 16 | 15 | 15 | | 15 | 14 | 14 |

| Intracerebral leucocytes* | Sample size behavior | | | Parameters flow cytometry | Sample size flow cytometry | | |
|---|---|---|---|---|---|---|---|
| | C | S | R | | C | S | R |
| Cohort 1 | 6 | 4 | 6 | CD11b, CD45 | 6 | 4 | 6 |
| Cohort 2 | 5 | 6 | 4 | CCR2, CD11b, CD11c, CD45, Ly6C | 5 | 4[d] | 4 |
| Cohort 3 | 5 | 5 | 5 | CCR2, CD11b, CD11c, CD45, Ly6C | 5 | 5 | 5 |
| Total | 16 | 15 | 15 | | 16 (11) | 13 (9) | 15 (9) |

* Same animals were used for the indicated flow cytometric analyses. [a] Unstimulated spleen cells were not investigated in this cohort. [b] The staining did not work for one sample in each group. [c] One cell pellet was lost during the staining process. [d] two samples were lost during Percoll gradient separation..
