# Supplementary material for: Alterations of the Innate Immune System in Susceptibility and Resilience After Social Defeat Stress
Source: Front Behav Neurosci. 2018 Jul 13;12:141. doi: 10.3389/fnbeh.2018.00141 (PMC6053497; doi:10.3389/fnbeh.2018.00141)
Supplement: Supplementary file 2 [file Table_2.PDF]

Supplementary Table 2. Overview of the statistical data for all analyzed parameters. C: control, S: susceptible, R: resilient.

Parameter	mean ± SEM			ANCOVA		Bonferroni posthoc		
	C	S	R			C vs. S	C vs. R	S vs. R
n	16	15	15					
Absolute cell number (x10^{7})	5.39 ± 0.94	13.64 ± 3.91	9.14 ± 2.45	F(2,41) = 4.47	**p = .018**	**p = .015**	p = .259	p = .723
n	10	11	9					
% CD11c^{+} MHC-II^{+}	3.5 ± 0.4	1.4 ± 0.2	1.6 ± 0.3	F(2,26) = 24.19	**p < .001**	**p < .001**	**p < .001**	p > .999
# CD11c^{+} MHC-II^{+} (x 10^{6})	1.01 ± 0.33	1.12 ± 0.34	0.96 ± 0.39	F(2,26) = 0.09	p = .913			
MFI of MHC-II (CD11c^{+} MHC-II^{+} pregate)	29123 ± 3106	45043 ± 5761	34711 ± 3352	F(2,26) = 6.99	**p = .004**	**p = .003**	p = .372	p = .171
MFI of CD80 (CD11c^{+} MHC-II^{+} pregate)	1591 ± 172	2024 ± 262	1664 ± 206	F(2,26) = 6.4972	**p = .005**	**p = .004**	p = .629	p = .125
% CD11c^{+} CD11b^{-} (CD11c^{+} MHC-II^{+} pregate)	31.4 ±1.0	42.1 ± 1.5	38.8 ± 1.4	F(2,26) = 22.92	**p < .001**	**p < .001**	**p < .001**	p = .277
# CD11c^{+} MHC-II^{+} CD11b^{-} (x10^{5})	3.29 ± 1.08	4.96 ± 1.51	3.93 ± 1.60	F(2,26) = 2.03	p = .151			
MFI of MHC-II (CD11c^{+} MHC-II^{+} CD11b^{-} pregate)	25641 ±2932	43256 ± 5658	33598 ± 3549	F(2,26) = 9.52	**p = .001**	**p = .001**	p = .096	p = .199
MFI of CD80 (CD11b^{-} CD11c^{+} MHC-II^{+} pregate)	1350 ± 196	1493 ± 217	1226 ± 140	F(2,26) = 0.78	p = .468			
% CD11c^{+} CD11b^{+} (CD11c^{+} MHC-II^{+} pregate)	67.8 ± 1.1	56.8 ± 1.5	60.0 ± 1.4	F(2,26) = 21.04	**p < .001**	**p < .001**	**p < .001**	p = .353
# CD11c^{+} MHC-II^{+} CD11b^{+} (x10^{5})	6.72 ± 2.24	6.15 ± 1.92	5.62 ± 2.24	F(2,26) = 0.68	p = .514			
MFI of MHC-II (CD11c^{+} MHC-II^{+} CD11b^{+} pregate)	30850 ±3215	46823 ±6183	35560 ± 3270	F(2,26) = 5.71	**p = .009**	**p = .008**	p = .681	p = .175
MFI of CD80 (CD11b^{+} CD11c^{+} MHC-II^{+} pregate)	1703 ± 169	2414 ±320	1950 ± 254	F(2,26) = 10.03	**p = .001**	**p < .001**	p = .121	p = .125
% CD11b^{+} CD11c^{-}	0.67 ± 0.05	2.2 ± 0.5	1.5 ± 0.2	F(2,26) = 5.21	**p = .013**	**p = .010**	p = .310	p = .517
# CD11b^{+} CD11c^{-} (x10^{5})	1.72 ± 0.56	16.77 ± 5.60	10.05 ± 4,28	F(2,26) = 6.14	**p = .007**	**p = .006**	p = .119	p = .783
% Ly6G^{hi} Ly6C^{low} (CD11b^{+} CD11c^{-} pregate)	53.2 ± 3.0	59.8 ± 2.4	64.0 ± 2.2	F(2,26) = 4.09	**p = .028**	p = .224	**p = .028**	p = .888
# CD11b^{+} CD11c^{-} Ly6G^{hi} Ly6C^{low} (x10^{4})	9.83 ± 3.24	89.24 ± 28.19	59.79 ± 25.28	F(2,26) = 7.04	**p = .004**	**p = .004**	**p = .048**	p > .999
% Ly6C^{hi} Ly6G^{low} (CD11b^{+} CD11c^{-} pregate)	19.5 ±1.6	24.9 ± 2.0	20.7 ± 2.1	F(2,26) = 2.14	p = .138			
# CD11b^{+} CD11c^{-} Ly6C^{hi} Ly6G^{low} (x10^{4})	3.07 ± 0.99	48.96 ± 18.60	23.82 ± 11.06	F(2,26) = 4.44	**p = .022**	**p = .019**	p = .421	p = .579

Supplementary Table 2 continued

Parameter	mean ± SEM			ANCOVA		Bonferroni posthoc		
	C	S	R			C vs. S	C vs. R	S vs. R
n	15	14	14					
% IL-12$^+$ (CD11c$^+$ pregate)	1.3 ± 0.1	1.8 ± 0.3	2.0 ± 0.3	F(2,38) = 3.85	**p = .030**	p = .137	**p = .039**	p > .999
# CD11c$^+$ IL-12$^+$ (x10^4)	3.50 ± 0.76	7.77 ±2.45	6.68 ± 1.64	F(2,38) = 2.85	p = .071			
% TNF$^+$ (CD11c$^+$ pregate)	2.4 ± 0.5	2.9 ± 0.4	2.2 ±0.5	F(2,38) = 0.21	p = .811			
# CD11c$^+$ TNF$^+$ (x10^4)	3.09 ± 0.98	8.09 ± 2.86	4.59 ± 1.70	F(2,38) = 2.57	p = .090			
% IL-12$^+$ (CD11b$^+$ CD11c$^-$ pregate)	1.3 ± 0.2	1.3 ± 0.2	1.4 ± 0.2	F(2,38) = 0.40	p = .675			
# CD11b$^+$ CD11c$^-$ IL-12$^+$ (x10^5)	0.96 ±0.25	1.41 ± 0.32	1.39 ± 0.30	F(2,38) = 2.37	p = .107			
% TNF$^+$ (CD11b$^+$ CD11c$^-$ pregate)	0.29 ± 0.04	0.53 ± 0.09	0.40 ± 0.06	F(2,38) = 3.59	**p = .037**	**p = .033**	p = .500	p = .680
# CD11b$^+$ CD11c$^-$ TNF$^+$ (x10^4)	1.73 ± 0.48	7.21 ± 2.42	3.84 ± 1.16	F(2,38) = 4.30	**p = .021**	**p = .017**	p = .640	p = .341
n	16	13	15					
% CD45^{int} CD11b$^+$	8.0 ± 1.7	10.2 ± 2.1	8.6 ± 1.5	F(2,39) = 1.17	p = .322			
% CD45^{hi} CD11b$^+$	4.9 ± 0.9	8.3 ± 1.6	7.5 ± 1.4	F(2,39) = 7.67	**p = .002**	**p = .001**	p = .053	p = .482
n	10	9	9					
% CD45^{hi} (vs. SSC-A)	19.8 ± 3.2	20.3 ± 3.9	14.3 ± 1.5	F(2,24) = 0.08	p = .920			
% CD11c$^+$ (CD45^{hi} pregate)	9.7 ± 0.9	15.9 ± 1.1	10.9 ± 2.0	F(2,24) = 13.31	**p < .001**	**p < .001**	**p = .004**	p = .639
% CCR2$^+$ Ly6C^{hi} (CD45^{hi} pregate)	6.8 ± 1.0	13.8 ± 3.0	19.5 ± 3.4	F(2,24) = 5.94	**p < .008**	**p = .007**	p = .151	p = .603
