# Supplementary material for: Alterations of the Innate Immune System in Susceptibility and Resilience After Social Defeat Stress
Source: Front Behav Neurosci. 2018 Jul 13;12:141. doi: 10.3389/fnbeh.2018.00141 (PMC6053497; doi:10.3389/fnbeh.2018.00141)
Supplement: Supplementary file 3 [file Table_3.PDF]

Supplementary Table 3. Explorative correlation analysis of parameters of the innate immune system and social avoidance behavior as indicator of stress vulnerability. Only variables that were either significantly changed in susceptible or in resilient mice and thus indicated a possible relationship with stress vulnerability were included in this analysis.

**Parameter**	**Interaction ratio**			**Interaction time (s)**		
	**r**	**p**	**N**	**r**	**p**	**n**
**A) Dendritic cells in the spleen**						
MFI of MHC-II ($CD11c^+$ $MHC\text{-}II^+$ pregate)	–.428	**.018**	30	–.491	**.006**	30
MFI of CD80 ($CD11c^+$ $MHC\text{-}II^+$ pregate)	–.362	**.049**	30	–.485	**.007**	30
MFI of MHC-II ($CD11b^-$ $CD11c^+$ $MHC\text{-}II^+$ pregate)	–.464	**.010**	30	–.521	**.003**	30
MFI of MHC-II ($CD11b^+$ $CD11c^+$ $MHC\text{-}II^+$ pregate)	–.393	**.032**	30	–.450	**.013**	30
MFI of CD80 ($CD11b^+$ $CD11c^+$ $MHC\text{-}II^+$ pregate)	–.467	**.009**	30	–.619	**< .001**	30
**B) Monocytes and neutrophils in the spleen**						
% $CD11b^+$ $CD11c^-$	–.361	.050	30	–.405	**.026**	30
# $CD11b^+$ $CD11c^-$ ($x10^5$)	–.371	**.044**	30	–.420	**.021**	30
% $Ly6G^{hi}$ $Ly6C^{low}$ ($CD11b^+$ $CD11c^-$ pregate)	–.143	.451	30	–.173	.360	30
# $CD11b^+$ $CD11c^-$ $Ly6C^{hi}$ $Ly6G^{low}$ ($x10^4$)	–.392	**.032**	30	–.430	**.018**	30
**C) Cytokine-production after LPS stimulation**						
% $IL\text{-}12^+$ ($CD11c^+$ pregate)	.014	.928	46	–.136	.366	46
% TNF^+ ($CD11b^+$ $CD11c^-$ pregate)	–.340	**.021**	46	–.382	**.009**	46
# TNF^+ $CD11b^+$ $CD11c^-$ ($x10^4$)	–.429	**.003**	46	–.484	**.001**	46
**D) Microglia and peripheral myeloid cells in the brain**						
% $CD45^{hi}$ $CD11b^+$	–.344	**.019**	46	–.349	**.017**	46
% $CCR2^+$ $Ly6C^{hi}$ ($CD45^{hi}$ pregate)	–.492	**.006**	30	–.454	**.012**	30
