# Supplementary material for: Alterations of the Innate Immune System in Susceptibility and Resilience After Social Defeat Stress
Source: Front Behav Neurosci. 2018 Jul 13;12:141. doi: 10.3389/fnbeh.2018.00141 (PMC6053497; doi:10.3389/fnbeh.2018.00141)
Supplement: Supplementary file 4 [file Image_1.pdf]

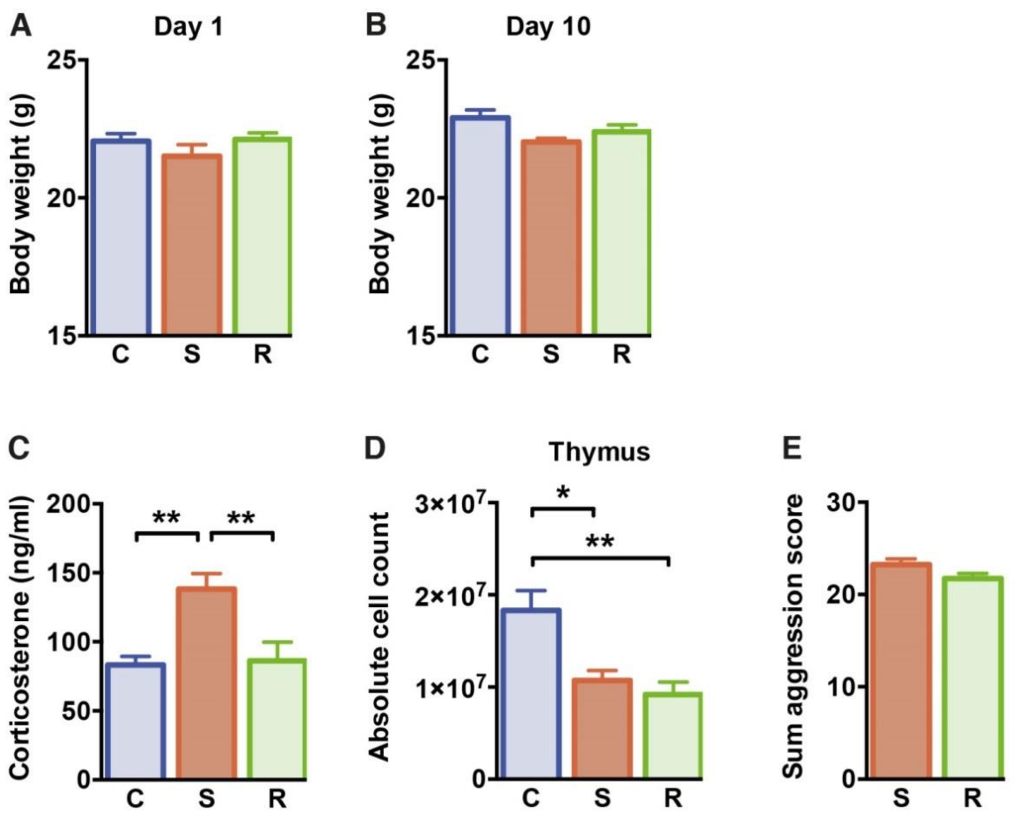

Supplementary Figure 1. A, B) Equivalent body weight of mice before and after 10 days of social defeat. During the 10 days of stress exposure mice of all groups gained about 0.5 to 1 g body weight. $n_C = 16$, $n_S = 15$, $n_R = 15$. C) Elevated corticosterone concentrations in the plasma of susceptible mice at day 12, two days after the end of the social defeat paradigm. $n_C = 13$, $n_S = 14$, $n_R = 7$. D) Decreased total numbers of thymocytes two days after 10 days of social defeat in susceptible and resilient mice. $n_C = 4$, $n_S = 5$, $n_R = 5$. E) The score of agonistic interactions did not differ between susceptible and resilient mice indicating that resilience did not result from differences in aversive experiences. $n_S = 14$, $n_R = 14$. *: $p < .05$, **: $p < .01$.

Supplementary material and methods

Corticosterone analysis

The day after the social interaction test, 13 control, 14 susceptible, and 7 resilient animals were sacrificed for blood collection and organ dissection. Animals were deeply anesthetized and the thorax opened. Blood was taken from the right ventricle before transcardial perfusion with ice-cold PBS, collected in EDTA prepared microtubes (Sarstedt, Nümbrecht, Germany) and centrifuged for 5 min at 2000 g. Plasma was taken and stored at –20°C until analysis. All blood samples were taken within a maximum of 3 min from touching the animal's cage. Corticosterone levels were assessed in duplicates from plasma samples as described before (Buschert et al., 2016) utilizing a commercially available ELISA kit (IBL International GmbH, Hamburg, Germany).

Thymus cell count

The thymus was dissected from 4 control, 5 susceptible and 5 resilient mice the day after the social interaction test, at day 12 of the experiment. A single cell suspension was obtained by pressing the tissue through a 100 µm cell strainer using a syringe plunger. Cells were counted using a CASY cell counter (Roche, Mannheim, Germany).

References

Buschert, J., Sakalem, M.E., Saffari, R., Hohoff, C., Rothermundt, M., Arolt, V., et al. (2016). Prenatal immune activation in mice blocks the effects of environmental enrichment on exploratory behavior and microglia density. *Prog Neuropsychopharmacol Biol Psychiatry* 67**,** 10-20. doi: 10.1016/j.pnpbp.2016.01.005.
