# Supplementary material for: Alterations of the Innate Immune System in Susceptibility and Resilience After Social Defeat Stress
Source: Front Behav Neurosci. 2018 Jul 13;12:141. doi: 10.3389/fnbeh.2018.00141 (PMC6053497; doi:10.3389/fnbeh.2018.00141)
Supplement: Supplementary file 5 [file Image_2.PDF]

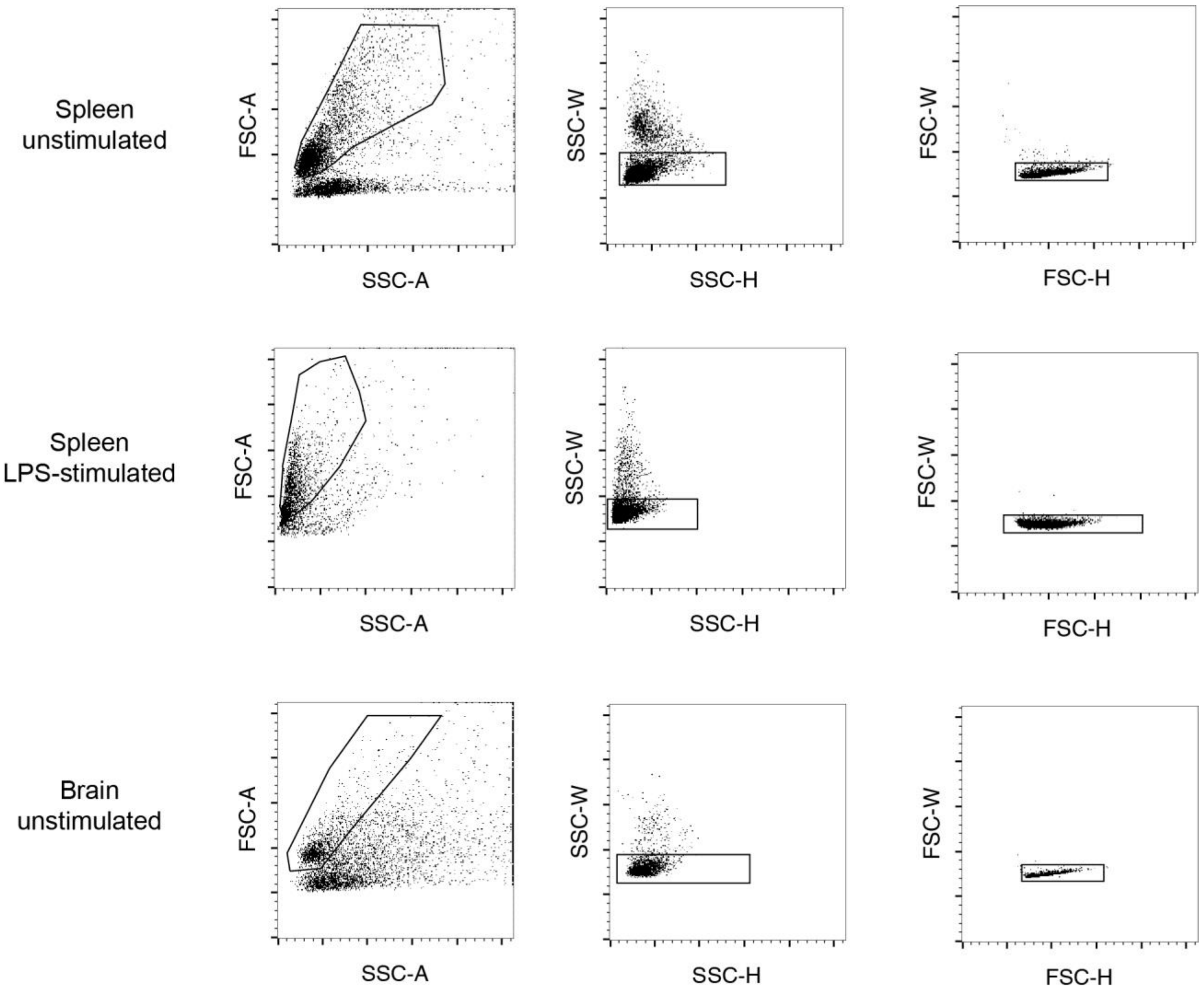

Supplementary Figure 2. Gating strategies for splenocytes and mononuclear cells isolated from the brain of experimental mice. Cells were life gated by FSC vs. SSC characteristics, single cells were gated using SSC and FSC Height (H) and Width (W) dot plots in all flow cytometry data analyses.
